# Special Issue: Molecular Advances in *Helicobacter pylori* Infections and Treatments

**DOI:** 10.3390/ijms27020778

**Published:** 2026-01-13

**Authors:** Dmitry S. Bordin

**Affiliations:** 1 Department of Pancreatic, Biliary and Upper Digestive Tract Disorders, A. S. Loginov Moscow Clinical Scientific Center, 111123 Moscow, Russia; dmitrybordin@gmail.com; Tel.: +7-495-304-3035 (ext. 1585); 2 Department of Propaedeutic of Internal Diseases and Gastroenterology, Russian University of Medicine, 127006 Moscow, Russia; 3 Department of General Medical Practice and Family Medicine, Tver State Medical University, 170100 Tver, Russia

*Helicobacter pylori* (*H. pylori*) infection is the leading cause of inflammatory changes in the gastric mucosa and a well-established carcinogen for gastric cancer. To foster interdisciplinary research into the pathogenesis of *H. pylori*–associated diseases, the European *Helicobacter pylori* Study Group (EHSG) was founded in 1987. Under its auspices, a series of consensus conferences bringing together leading experts were held to develop evidence-based approaches to diagnosis and treatment. Over recent decades, molecular and genetic studies have become indispensable for the development of highly accurate diagnostic tests for *H. pylori* infection, for elucidating key mechanisms underlying bacterium-induced inflammation and its persistence, for advancing research into gastric carcinogenesis, and for exploring the extra-gastrointestinal effects of the bacterium.

In the review Bordin D.S. et al, the authors discuss molecular mechanisms linking autoimmune gastritis (AIG) and *Helicobacter pylori* infection—two leading etiological drivers of chronic atrophic gastritis and key contributors to the development of gastric atrophy and intestinal metaplasia. The review outlines several interaction scenarios: AIG without *H. pylori* infection (“pure” AIG), co-existence of AIG and *H. pylori*, and AIG observed in the post-eradication period (contribution 1).

Importantly, in patients with AIG, the prognostic value of the Operative Link on Gastritis Assessment (OLGA) and the Operative Link on Gastric Intestinal Metaplasia Assessment (OLGIM) systems for predicting epithelial gastric neoplasia warrants further investigation. Because primary AIG typically spares the antrum, the OLGA stage in such patients generally does not exceed stage II, which may limit the discriminative performance of OLGA staging in “pure” autoimmune disease.

The RE.GA.IN consensus statement [1] notes that OLGA stages III–IV in a patient with AIG provide strong evidence of a previous *H. pylori* infection, which may have induced atrophic changes in the antrum and thereby “upstaged” OLGA.

Considering the molecular mechanisms of atrophy in both isolated AIG and AIG occurring in the setting of concurrent or past *H. pylori* infection supports a more personalized assessment of gastric cancer risk and helps disentangle the relative contribution of each etiological factor to carcinogenesis.

Accounting for the simultaneous (or sequential) effects of AIG and *H. pylori* is crucial for accurate diagnostic reasoning, because the gastritis phenotype, the topography of atrophy/metaplasia, and the interpretation of OLGA/OLGIM staging may differ substantially between “pure” AIG and AIG associated with current or previous *H. pylori* infection. In clinical practice, this affects not only risk stratification (including cancer vigilance) but also management decisions: the need to (re-)verify *H. pylori* status and perform eradication therapy when infection is detected, as well as subsequent follow-up, given that autoimmune pathology may manifest or progress after eradication and can be masked by active *H. pylori* gastritis prior to treatment.

Recent evidence on diagnostic criteria and endoscopic/histological features of AIG underscores the importance of a standardized approach to recognizing AIG, particularly in situations where it may overlap with *H. pylori*–associated inflammation [2]. Additional clinical observations also suggest that rapid progression of AIG may occur after *H. pylori* eradication in a subset of patients, highlighting the need for careful discussion and incorporation into monitoring recommendations [3]. Finally, reports describing the manifestation (“unmasking”) of AIG after *H. pylori* eradication emphasize that post-eradication mucosal changes should be interpreted within a two-factor framework, avoiding an overly simplistic attribution of gastritis to a single cause in an individual patient [4].

The first statement of the Maastricht VI consensus emphasizes that *H. pylori* invariably induces gastric mucosal inflammation (i.e., gastritis), which may progress to clinically significant complications—including peptic ulcer disease, gastric cancer, and MALT lymphoma—regardless of the presence or absence of symptoms [5].

In an experimental study, Zhang X. et al. provide new molecular evidence supporting a dynamic cellular and immunological adaptation of gastric epithelial cells to chronic *H. pylori* infection (contribution 2). Using the human gastric epithelial cell line GES-1 exposed to *H. pylori* lysate, the authors modelled the temporal trajectory of epithelial injury and adaptation under infection-related stimuli. Acute exposure resulted in marked morphological and functional alterations, including suppression of proliferation and autophagy, with concomitant induction of apoptosis. In contrast, during chronic *H. pylori* exposure, the epithelial response shifted from inflammation-driven apoptosis toward adaptive survival mechanisms. In vivo, *H. pylori* lysates initially increased TLR4/5/9 expression and triggered immune-cell activation with enhanced secretion of pro-inflammatory cytokines; however, prolonged *H. pylori* persistence, conversely, reduced TLR expression, thereby facilitating continued bacterial colonization and progression of gastric mucosal atrophy. Overall, these findings add to current concepts of the dynamic cellular changes that may underlie *H. pylori*–driven epithelial transformation along the pathway to gastric cancer.

Despite major advances in the diagnosis and treatment of *H. pylori* infection over recent decades, the timely detection of the bacterium using highly informative, non-invasive tests remains an important unmet need—particularly in pediatric practice [6,7]. An innovative diagnostic approach for *H. pylori* infection in children is explored in the study by Zakrzewski M. et al. (contribution 3). Saliva, as a substrate for detecting bacterial metabolites and host-derived predictors of ongoing gastrointestinal inflammation related to *H. pylori*, represents a promising target for further research. In that study, the authors evaluated salivary concentrations of selected inflammatory markers (TNF-α, IL-8) alongside additional biomarkers (neutrophil defensin-1, sICAM-1, calprotectin, matrix metalloproteinase-9, matrix metalloproteinase-2, lactoferrin, and TLR-2). The reported results open new perspectives for understanding the pathogenic mechanisms of oral inflammatory changes in the setting of *H. pylori* infection and for improving non-invasive *H. pylori* diagnostics in children.

Saliva should be recognized as a valuable diagnostic medium across a wide range of conditions, including digestive diseases (e.g., gastroesophageal reflux disease, GERD) as well as systemic disorders such as diabetes mellitus, hepatitis, oral diseases, and immunodeficiency-related conditions.

Another study included in this Special Issue addresses the prevention of *H. pylori* infection of the gastric mucosa. The oral cavity may serve as a reservoir for *H. pylori*, with potential links to both dental disease and gastrointestinal pathology [8,9]. In an experimental setting, Chen X. et al. evaluated the antibacterial and antibiofilm activity of dimethylaminododecyl methacrylate (DMADDM) and DMADDM-modified giomer restorative materials against *H. pylori* (contribution 4). Importantly, DMADDM can copolymerize with dental resins, preventing burst release and rapid loss of the active antibacterial component, while enabling a potentially durable, contact-killing bactericidal effect of restorative dental materials against *H. pylori*. In vivo experiments further supported that pretreatment with a DMADDM-modified dental resin effectively reduced gastric colonization by bacteria originating from the oral cavity. Overall, this work contributes to the development of preventive strategies against *H. pylori*.

While promising, this preventive approach also warrants a deeper analysis of possible modulation of the oral and gastric microbiota, particularly in the context of the protective roles of indigenous microbial communities and their metabolites across a broad spectrum of digestive diseases. It is increasingly appreciated that the stomach harbors a well-adapted, niche-specific microbial community whose composition is shaped by the physiological conditions of this unique ecological environment.

Regarding extra-gastroduodenal manifestations of *H. pylori* infection, the available evidence for causal relationships remains insufficient [5,10]. In a Swedish case–control study by Wärme J. et al., the association between *H. pylori* infection and inflammatory protein biomarkers was investigated in patients with myocardial infarction with obstructive coronary artery disease (MI-CAD) and myocardial infarction with non-obstructive coronary arteries (MINOCA) (contribution 5). The authors hypothesized that *H. pylori* may contribute to systemic inflammation and thus be linked to cardiovascular disease. In their cohort, *H. pylori* infection was detected in approximately one in five patients with myocardial infarction, irrespective of coronary obstruction status. Among those who tested positive for *H. pylori*, 45.5% also had antibodies against CagA. The proportion of individuals with antibodies to *H. pylori* and CagA was higher in the MINOCA group compared with the MI-CAD group and healthy controls (60.0%, 31.6%, and 43.8%, respectively), although the difference did not reach statistical significance. *H. pylori*–positive subjects exhibited higher levels of inflammatory proteins, suggesting a potential role of infection in systemic inflammatory responses and a possible contribution to cardiovascular pathology. In an adjusted model, seven proteins were associated with *H. pylori*: tissue plasminogen activator (tPA), interleukin-6 (IL-6), myeloperoxidase (MPO), TNF-related activation-induced cytokine (TRANCE), pappalysin-1 (PAPPA), soluble urokinase plasminogen activator receptor (suPAR), and P-selectin glycoprotein ligand-1 (PSGL-1).

*H. pylori* is a globally prevalent gastrointestinal pathogen that is increasingly linked to a range of extra-gastric chronic non-communicable diseases. In cardiovascular disease, *H. pylori* has been proposed to aggravate chronic vascular inflammation, endothelial dysfunction, and autoimmune responses. Although causality has not been fully established, these observations raise the possibility that *H. pylori* could represent a modifiable risk factor for certain non-communicable conditions. Further longitudinal and interventional studies are needed to determine whether *H. pylori* eradication can meaningfully reduce the burden of cardiovascular disease.

In closing, I would like to thank all authors who contributed to this Special Issue on molecular advances in *H. pylori* infections and treatments.
